# Joint position on vitamin D prescription in the adult Mexican population by AMMOM, AMEC, AMG, CMIM, CMO, CMR, CONAMEGER, FEMECOG, and FEMECOT

**DOI:** 10.1007/s11657-025-01563-y

**Published:** 2025-06-13

**Authors:** Jose Francisco Torres-Naranjo, Hugo Gutierrez-Hermosillo, Pedro Alberto Garcia-Hernandez, Roberto E. López Cervantes, Hilario E. Avila Armengol, Rafael Bedoya Torres, Alhelí Lucía Bremer Aztudillo, Juan Humberto Medina Chávez, Rocio Morales Delgado, Eva M. Perusquía Frías, Alan Rios Espinosa, Alejandro Vázquez Alanís

**Affiliations:** 1 Centro de Investigación Ósea y de la Composición Corporal (CIO), Guadalajara, México; 2 Escuela Nacional de Estudios Superiores, Unidad Leon, Universidad Nacional Autonoma de México, Mexico City, México; 3 Universidad Autónoma de Nuevo León, San Nicolás de los Garza, México; 4 Federación Mexicana de Colegios de Ortopedia y Traumatologia, Guadalajara, México; 5 Colegio Mexicano de Reumatología, Mexico City, México; 6 Federación Mexicana de Colegios de Obstetricia Y Ginecología. A. C., Ciudad de México, CDMX, México; 7 Facultad de Medicina y Psicología, Universidad Autónoma de Baja California, Tijuana, México; 8 Academia Mexicana de Geriatría, Mexico City, México; 9 Geriatric Service, Hospital Universitario “Dr. José E. González”, Universidad Autónoma De Nuevo León, San Nicolás de los Garza, México; 10 Colegio De Medicina Interna De México, Mexico City, México; 11 Asociación Mexicana para el Estudio del Climaterio, Mexico City, México; 12 Asociación Mexicana de Metabolismo Óseo Y Mineral (AMMOM), Santiago de Querétaro, México

**Keywords:** Vitamin D, Supplementation, Hypovitaminosis, 25(OH)D

## Abstract

**Background:**

Vitamin D deficiency remains a critical health concern linked to skeletal disorders such as osteoporosis, osteomalacia, and fractures. Recent evidence highlights the broader role of vitamin D in preventing chronic conditions, including autoimmune diseases, diabetes, and cardiovascular events. However, inconsistencies in clinical practice across Mexico and limited population-specific data necessitate standardized guidelines to address diagnostic and therapeutic challenges.

**Objective:**

To establish evidence-based recommendations for diagnosing and prescribing vitamin D supplements tailored to the Mexican adult population, reducing practice variability while promoting optimal health outcomes.

**Methods:**

A multidisciplinary panel comprising specialists from nine leading Mexican medical organizations conducted a consensus process using the Delphi methodology. The recommendations were developed using a combined approach, integrating extensive literature reviews with expert consensus to address areas where empirical evidence is limited. The process informed guidelines for vitamin D supplementation, measurement criteria, and therapeutic monitoring.

**Results:**

Key recommendations include: Measuring 25(OH)D levels in adults with risk factors or conditions associated with hypovitaminosis D, avoiding routine screening in healthy individuals. Defining vitamin D deficiency as < 20 ng/mL, insufficiency as 20–29 ng/mL, and sufficiency as 30–100 ng/mL. Preferring cholecalciferol for supplementation, with calcifediol reserved for specific cases requiring rapid correction or compromised hepatic hydroxylation. Regularly monitor serum 25(OH)D concentrations to achieve and maintain levels between 30 and 60 ng/mL, ensuring safety and therapeutic efficacy.

**Conclusion:**

This joint position provides a comprehensive framework for managing hypovitaminosis D in Mexican adults. The recommendations aim to harmonize clinical practices, improve patient outcomes, and inform public health strategies for equitable resource allocation. Ongoing evaluation and stakeholder feedback will ensure adaptability and relevance as new evidence emerges.

## Introduction

Vitamin D is a fat-soluble nutrient and a prohormone that plays a significant role in multiple physiological processes within a complex hormonal system. This system is crucial for numerous well-characterized physiological functions. Due to its fundamental involvement in calcium homeostasis, hypovitaminosis D is associated with skeletal disorders such as rickets, osteomalacia, and osteoporosis.

Our understanding of vitamin D has significantly evolved in recent decades, broadening our perspective beyond its traditional role. Recent research has sparked growing interest in its interaction with other body systems. Explorations into the effects of vitamin D supplementation have encompassed various areas of health, including its impact on the musculoskeletal and immune systems and its influence on chronic diseases such as osteoporosis, sarcopenia, type 2 diabetes, myocardial infarction, and various types of cancer.

The widespread use of vitamin D supplementation, in the form of dietary sources and appropriate prescriptions, has substantially reduced the incidence of rickets, making it a rare disease among children in Mexico. However, many adults suffer from vitamin D deficiency. Consequently, they often receive pharmacological vitamin D compounds. Despite extensive research promoting diverse approaches to vitamin D supplementation that consider individual patient needs and the complexities of healthcare delivery, challenges persist in Mexico. These challenges include unwarranted variation, disparities in care, and inefficiencies. Therefore, it is crucial to establish greater consensus on optimal dosages and supplementation protocols to optimize therapeutic outcomes while minimizing potential risks across various clinical scenarios.

## Objective

This joint statement aims to provide updated guidance diagnosing hypovitaminosis D and prescribing therapeutic vitamin D supplements in the Mexican adult population while mitigating heterogeneity in clinical practice nationwide.

## Methodology

The Mexican Association of Bone and Mineral Metabolism (AMMOM), along with other national organizations, namely, the Mexican Association for the Study of Climacteric (AMEC), Mexican Academy of Geriatrics (AMG), the College of Internal Medicine of Mexico (CMIM), the Mexican College of Orthopedics and Traumatology (CMO), the Mexican College of Rheumatology (CMR), the National College of Geriatric Medicine (CONAMEGER), the Mexican Federation of Obstetrics and Gynecology Colleges (FEMECOG), and the Mexican Federation of Orthopedics and Traumatology Colleges (FEMECOT), jointly convened an expert panel. This panel consists of specialists from various disciplines in the clinical aspects of vitamin D, including endocrinologists, gynecologists, geriatricians, internists, orthopedic surgeons, and rheumatologists. The primary focus was establishing a position statement in response to the widespread use and potential overuse of vitamin D, by general practitioners and specialists.

Panel members were selected based on their knowledge and experience in the field of vitamin D and their ability to assess and analyze existing published information for clinical decision-making. Before inclusion in the expert panel, all members disclosed potential conflicts of interest. Expert panel members did not receive financial compensation during the analysis or preparation of the document.

The panel was urged to conduct exhaustive literature reviews to address these aspects comprehensively and thoroughly explore the topics covered in this consensus document. Identifying gaps in existing evidence was an essential step in driving the expert consensus process. The Delphi methodology, with four rounds of evaluation, was employed to reach a consensus opinion. In this process, a consensus criterion was established, requiring a minimum of 70% agreement to validate the conclusions. This methodological approach ensures the rigor and representativeness of the consensus opinions, grounding the recommendations in the critical review of scientific literature and the collective expertise of experts. Essential lineaments that guided the recommendations prioritize transparency, flexibility, inclusivity, clarity, and feasibility to ensure that all stakeholders can understand and implement them effectively.

## Position statements and considerations

Below, we describe the consensus statements accompanied by relevant considerations. The direction of this work was based on evidence available up to 2023, and the expert panel was urged to conduct exhaustive literature reviews on the topics covered in this consensus document.

### Who should receive therapeutic vitamin D supplementation

Pharmacological vitamin D supplementation is recommended for adults with documented hypovitaminosis D, defined as serum 25-hydroxyvitamin D [25(OH)D] levels below 30 ng/mL (< 75 nmol/L).

Pharmacological vitamin D supplementation should be established only in patients with suboptimal levels of 25(OH)D. While widespread prescription or consumption of pharmacological vitamin D supplements without specific medical indication should not be promoted [15].

Pharmacological vitamin D supplementation in adults with low baseline 25(OH)D levels has shown positive effects on individual health. Benefits have been observed in relation to rickets [23], osteomalacia, osteoporosis, and mineral metabolism. Regarding non-musculoskeletal health outcomes, there is evidence suggesting that pharmacological vitamin D supplementation could have benefits in preventing acute respiratory infections [54], cancer mortality [47], diabetes mellitus prevention [40], and recently, reducing some autoimmune diseases [19], as well as other conditions; however, this panel considers there is no conclusive evidence.

It is important to note that while assessing 25(OH)D levels before initiating supplementation, in cases of fragility fractures, especially hip fractures, where vitamin D deficiency may hinder recovery, supplementation may be necessary even before obtaining laboratory results [49]. This is especially relevant in institutions where 25(OH)D measurement is not available. In such cases, the benefit of supplementation may outweigh the risk of not having accurate laboratory data immediately.

Consider that the main risk of excessive vitamin D supplementation is hypervitaminosis, extremely sporadic and generally associated with excessive supplement consumption over long periods, or the lack of distinction between calcitriol, calcifediol, and cholecalciferol, which can lead to hypercalcemia with symptoms such as nausea, vomiting, abdominal pain, hypercalciuria, and complications such as bone loss and renal insufficiency.

### When 25(OH)D levels measurement is recommended

Baseline 25(OH)D levels should be measured in adults before initiating pharmacological vitamin D supplementation.

Accurate determination of 25(OH)D blood levels is essential before starting pharmacological vitamin D supplementation in adults [10]. Measurement of 25(OH)D levels before pharmacological supplementation allows determining whether an individual has hypovitaminosis D and establishing the appropriate therapeutic dosage. In this context, determining 25(OH)D in the blood is the most appropriate way to diagnose hypovitaminosis D in adults, as it reflects both intake, endogenous production, and body reserves.

Performing measurements before and during supplementation provides more precise control and allows for rigorous treatment monitoring and adjustments as necessary. In cases where the assessment of serum 25(OH)D concentration is not possible in high-risk groups, vitamin D supplementation should be carried out according to the recommendations established for the general population, respecting the maximum doses for the corresponding age group [35].

Pharmacological vitamin D supplementation should not be indicated in healthy adults with 25(OH)D values within the optimal parameters.

#### Optimal concentrations and intervention thresholds

It is recommended that in adults, vitamin D deficiency be defined as a 25(OH)D level of less than 20 ng/mL (< 50 nmol/L), insufficiency as a 25(OH)D level of 20 to 29 ng/mL (50–< 75 nmol/L), and sufficiency as a 25(OH)D level of 30 to 100 ng/mL (75–250 nmol/L) Table 1 [12, 33].

**Table 1:** Vitamin D status based on 25(OH)D levels in adults

Vitamin D status	Serum 25(OH)D concentration	
Sufficiency	30 a 100 ng/mL	(75–250 nmol/L)
Insufficiency	20 a 29 ng/mL	(50–< 75 nmol/L)
Deficiency	< 20 ng/mL	(< 50 nmol/L)

Levels < 20 ng/mL ($\le$ 50 nmol/L) are widely used by researchers and available guidelines as indicative of deficiency and have been associated with increased risk of fractures, cardiovascular disease, colorectal cancer, diabetes, depressed mood, cognitive impairment, and mortality [22]. Association with falls has been observed in studies of institutionalized elderly populations [36]. There is debate about whether levels of 20–30 ng/mL (50–75 nmol/L) represent a deficiency, as levels > 24 ng/mL (> 60 nmol/L) have been associated with lower cardiovascular disease risk. Levels > 30 ng/mL (> 75 nmol/L) are associated with lower mortality and colorectal cancer risk [44]. Conflicting data exist on whether levels > 30 ng/mL (> 75 nmol/L) are associated with lower fracture risk.

Given the diverse viewpoints on optimal concentrations of 25(OH)D in adults, this panel recommends defining vitamin D deficiency as a 25(OH)D level below 20 ng/mL (< 50 nmol/L), insufficiency as a 25(OH)D level of 20 to 29 ng/mL (50–< 75 nmol/L), and sufficiency as a 25(OH)D level of 30 to 100 ng/mL (75–250 nmol/L). Although the suggested thresholds may be slightly higher at 30 ng/mL (75 nmol/L) for non-skeletal benefits, this aspect still requires study, so no specific recommendation is issued.

There is total consensus among the experts in this panel that levels below 20 ng/mL (< 50 nmol/L) are associated with bone health conditions, so any patient with values below this figure should receive pharmacological supplementation.

Considering the absence of a safe upper limit in current evidence, this panel recommends not exceeding 100 ng/mL serum levels and preferably maintaining levels between 30 and 60 ng/mL, a range where most evidence converges.

#### Whom to measure 25(OH)D levels

Measurement of baseline 25(OH)D levels is recommended in adults with risk factors or clinical conditions associated with hypovitaminosis D.

25(OH)D level measurement in the adult population is not recommended as a general screening method. Instead, it is suggested that 25(OH)D measurement be performed in adults with risk factors or clinical conditions associated with hypovitaminosis D. This recommendation extends to patients receiving vitamin D formulations as part of routine treatment (e.g., osteoporosis), for whom determining 25(OH)D levels is advised to appropriately adjust supplementation.

This panel suggests determining serum 25(OH)D levels in the following conditions associated with hypovitaminosis D [4–6, 13, 19, 20, 29, 34, 38, 39, 46, 48, 52, 53], (Table 2):

**Table 2:** Indications for measuring 25(OH)D in adults

*Musculoskeletal Disorders*	$\bullet$ Osteoporosis.
	$\bullet$ Osteopenia.
	$\bullet$ Low-energy trauma bone fractures.
	$\bullet$ Recurrent falls.
	$\bullet$ Sarcopenia.
	$\bullet$ Frailty Syndrome
*Endocrine and Metabolic Diseases/Conditions*	$\bullet$ Diabetes mellitus (type 1 and 2).
	$\bullet$ Metabolic syndrome.
	$\bullet$ Obesity.
	$\bullet$ Hyper- and hypoparathyroidism.
	$\bullet$ Hyper- and hypothyroidism.
	$\bullet$ Hypercalcemia and hypocalcemia.
	$\bullet$ Hypercalciuria.
	$\bullet$ Hyperphosphatemia.
	$\bullet$ Hyper- and hypophosphatasia.
	$\bullet$ Phosphaturia
*Malabsorption Syndromes*	$\bullet$ Exocrine pancreatic insufficiency.
	$\bullet$ Inflammatory bowel disease (Crohn’s disease, ulcerative colitis).
	$\bullet$ Cystic fibrosis.
	$\bullet$ Celiac disease.
	$\bullet$ Bariatric surgery.
	$\bullet$ Radiation enteritis
*Liver and Biliary Tract Diseases*	$\bullet$ Liver failure.
	$\bullet$ Pancreatic insufficiency
*Renal Diseases*	$\bullet$ Chronic kidney disease (especially stages 3–5)
*Skin Diseases*	$\bullet$ Atopic dermatitis.
	$\bullet$ Psoriasis
*Reduced Vitamin D Production in the Skin*	$\bullet$ Advanced age (especially > 70 years).
	$\bullet$ Use of sunscreen.
	$\bullet$ Habitual full-body clothing
*Long-term Medication Use*	$\bullet$ Antiepileptic drugs (e.g., valproate, phenytoin).
	$\bullet$ Antiretroviral medications.
	$\bullet$ Glucocorticoids.
	$\bullet$ Systemic antifungal medications.
	$\bullet$ Rifampicin.
	$\bullet$ Bile acid sequestrants (cholestyramine).
	$\bullet$ Lipase inhibitors (orlistat).
	$\bullet$ Polypharmacy
*Psychiatric and Central Nervous System Conditions*	$\bullet$ Major neurocognitive disorders.
	$\bullet$ Parkinson’s disease.
	$\bullet$ Anorexia nervosa
*Rheumatological, Granulomatous, and Neoplastic Diseases*	$\bullet$ Rheumatoid arthritis.
	$\bullet$ Systemic lupus erythematosus.
	$\bullet$ Multiple sclerosis.
	$\bullet$ Sarcoidosis.
	$\bullet$ Lymphomas

*These are key indications for measuring 25(OH)D. Assessing 25(OH)D levels in patients with clinical conditions associated with hypovitaminosis D is critical. While this list covers a wide range of conditions* [4–6, 13, 19, 20, 29, 34, 38, 39, 46, 48, 52, 53], *other clinical manifestations may also warrant 25(OH)D testing, emphasizing the need for clinical judgment in individual cases.*

### How to perform medical vitamin D supplementation in adults with hypovitaminosis D

Vitamin D formulation and dosing should be established based on serum 25(OH)D concentration, clinical conditions, previous prophylactic schemes, available formulations, and patient preferences [2].

Age, gender, ethnicity, body mass index, weight, gestational status, body composition, baseline 25(OH)D, and genetic factors may influence the response to vitamin D intake and supplementation. It should be considered in all adult patients undergoing medical vitamin D supplementation in any form [28]. Medical vitamin D supplementation should be implemented and monitored based on serum 25(OH)D concentrations in order to achieve and maintain the optimal concentration [35, 43, 51, 55].

#### Formulation selection

It is recommended that schemes for preventing and treating vitamin D deficiency in adults in Mexico be based on using cholecalciferol as the first-line option or calcifediol in specific medical conditions [32].

Supplementation with vitamin D2 (ergocalciferol) results in a lower increase in plasma 25(OH)D compared to D3 (cholecalciferol) per unit of administered vitamin D. Therefore, vitamin D3 is advised for medical vitamin D supplementation [3, 21, 31].

Cholecalciferol is also recommended as the first option for both prophylactic and treatment options. There are differences in the response to supplementation between cholecalciferol and calcifediol, due to higher absorption fraction and differences in tissue distribution of calcifediol compared to cholecalciferol, as well as hydroxylation of vitamin D3 to metabolites other than 25(OH)D [1, 7, 8].

Calcifediol should be used as a second option when cholecalciferol does not improve serum 25(OH)D concentration, an immediate increase in serum 25(OH)D concentration is required, or hepatic hydroxylation capacity is decreased [9, 11, 14].

#### Presentation and dosage

In agreement with the patient, the physician should select the appropriate presentation and dosage of vitamin D for each situation and ensure that the patient and caregivers understand the administration and monitoring regimen [18].

The physician should select the appropriate medication presentation for each situation and ensure that the prescription clearly indicates both the presentation and the dosage per intake and frequency of administration, in addition to clearly explaining the administration regimen of the prescribed medication to the patient and/or caregivers and ensuring that it has been understood correctly. The follow-up plan should also be agreed upon with the patient and caregiver [50].

Different formulations of vitamin D available in Mexico include cholecalciferol in presentations of 200 IU, 400 IU, 800 IU, 1600 IU in tablets, 2000 IU in chewable tablets, 4000 IU in dispersible tablets, 5000 IU in gel caps and tablets, 5600 IU, 25,000 IU, 50,000 IU, and 100,000 IU in gel caps, as well as calcifediol in a presentation of 0.266 mg in soft capsules and calcitriol in capsules of 0.25 mcg. These should be prescribed according to specific medical guidelines for each patient, presentation, and clinical situation. Adjusting the presentation and dosage based on the patient’s preference for daily, weekly, or monthly therapy may positively influence adherence.

The process of determining the appropriate dosage for vitamin D replacement involves balancing the need to raise serum 25-hydroxyvitamin D levels with consideration of body weight and baseline 25(OH)D concentrations. Notably, larger doses are often required for individuals with higher body weights, while those with lower baseline 25(OH)D levels exhibit more significant increases in response to supplementation. Understanding these factors is essential for healthcare providers to tailor replacement regimens effectively.

In contemporary clinical practice, standard protocols recommend administering vitamin D supplements on a monthly, weekly, or daily basis. However, individualized approaches may be necessary in cases of suspected malabsorption or poor treatment adherence. Additionally, predictive equations like those proposed by Singh and Bonham [45] or van Groningen et al. [17] can be valuable tools for guiding dose selection.

For hypovitaminosis D, we suggest a daily intake of 6000 IU or its equivalent, either weekly, monthly, or bimonthly for three months. After this initial phase, measure 25-hydroxivitamin D levels. If they fall between 30 and 60 ng/mL, continue with a dosage ranging from 600 to 4000 IU, considering higher doses for individuals with conditions such as obesity or those taking medications that interfere with vitamin D levels.

Formulas to calculate the bolus dose for hypovitaminosis D, such as the one proposed by Van Groningen et al. [17], can be employed if the physician opts for a more personalized approach to initiating oral vitamin D supplementation; therefore, according to this formula in a male patient with 15 ng/ml serum 25(OH) vitamin D, would need 144,000 UI as an initial dose if his weight is 60 kg (75–15) × 40 × 60), but 240,000 UI if his weight is 100 kg (75–15) × 40 × 100). Caution should be exercised with any formula used. While evidence suggests that boluses as large as 300,000 IU taken at once can be safe, it is advisable not to prescribe them, especially in older adults.

Clinicians must exercise caution and vigilance when prescribing vitamin D replacement therapy. They should explain the risks associated with vitamin D overdose and provide instructions on symptoms suggestive of overdose. Subsequent medical visits should confirm that the product is being administered correctly.

While aiming to achieve serum 25(OH)D levels above 30 ng/mL, they must be mindful of potential assay variability and individual patient factors. Maintaining serum levels within an target 25(OH)D range is crucial for ensuring therapeutic efficacy while minimizing the risk of toxicity.

Treatment should be continued for three months or until a stable, optimal serum 25(OH)D concentration is achieved. If clinical conditions associated with hypovitaminosis D persist, maintenance doses should be initiated.

### Evaluation of clinical conditions

Clinical conditions that interfere with the metabolism and actions of vitamin D and its metabolites should be evaluated in all adult patients with hypovitaminosis D [10].

Since various clinical conditions can affect vitamin D metabolism, its action pathways, and tissue response, a thorough medical history should be obtained before supplementation [26]. The presence of these conditions should be evaluated, and if necessary, relevant tests should be performed. Subsequently, the prescription should be adjusted according to the specific clinical condition of each patient.

Patients with chronic kidney disease, especially those on dialysis or in the end stage, are at risk of inadequate vitamin D activation, so complementary pharmacological supplementation with calcitriol should be considered [4, 46].

Serum 25 (OH) D and calcium concentrations should be carefully monitored in patients with chronic granuloma-forming disorders such as sarcoidosis, tuberculosis, and chronic fungal infections and in patients with lymphoma.

Whenever feasible, the risk of hypersensitivity to vitamin D (hypercalciuria, hypercalcemia, nephrolithiasis, nephrocalcinosis, or a history of other types of hypersensitivity to vitamin D in an individual or their relatives) should be evaluated before starting supplementation [16, 30].

In patients with skeletal symptoms, bone metabolic disease, and bone mineral disorders (bone deformities, bone pain, nonspecific musculoskeletal symptoms, fatigue syndrome, and a history of fragility fractures), calcium-phosphate metabolism parameters (Ca, PO4, ALP, PTH, calcium/creatinine ratio in urine) must be evaluated and monitored. Likewise, bone mineral density can be determined using bone DXA densitometry.

For some patients with chronic diseases (obesity, malabsorption syndromes, liver diseases, chronic inflammatory diseases) or taking medications that interfere with hepatic cytochrome P450 (i.e., glucocorticoids, anticonvulsants, antiretroviral drugs, or anticancer drugs), or rapid restoration of vitamin D deficiency might be necessary, the use of calcifediol in therapeutic doses of 0.266 µg monthly or more frequent is reasonable and justified.

Individuals who are overweight or obese require special attention, as these conditions generally require a higher dose of cholecalciferol compared to the recommended doses for individuals of the same age with normal body weight. In these individuals, calcifediol may be considered as an alternative treatment regimen [24].

### How to evaluate therapeutic response

It is recommended that 25(OH)D levels in adults receiving medical vitamin D supplementation be monitored.

Measurement of 25(OH)D levels is essential to evaluate the response to the therapeutic intervention in adults receiving medical vitamin D supplementation. Three months after initiating treatment, follow-up measurements of 25(OH)D levels are advised, and they should continue until target values are reached.

Monitoring is crucial to determining whether the prescription is adequate and ensuring therapeutic goals are achieved [37]. The time between starting vitamin D supplementation and stabilizing the new plasma 25(OH)D concentration ranges from 6 to 16 weeks. Therefore, samples measuring 25(OH)D concentration must be taken after sufficient time to reach the new steady state to assess the dose-response relationship [42]. Since calcitriol’s plasma half-life is very short, measuring plasma 25(OH)D concentration provides the most useful information for assessing bioavailability and response to vitamin D administration [25, 27].

The results of the 25 (OH) D test may vary depending on the assay method used, so follow-up measurements with the same assay are recommended whenever possible. We encourage the use of only validated assay methods.

#### Treatment adjustment

Vitamin D supplementation must be adjusted to achieve the therapeutic goal or if 25(OH)D levels exceed established limits.

Adjusting or replacing the vitamin D formulation is crucial to achieving the therapeutic goal or in case of an increase in 25(OH)D levels above established limits.

In all cases, it is essential to verify whether the previous treatment regimen (dosage, compliance, type of preparation) has been followed correctly and yielded appropriate results. This is imperative because it is possible, albeit remote, that the observed 25(OH)D levels have been achieved even with low patient compliance or that the patient has adjusted or replaced the dosage on their initiative.

*Concentrations of 25(OH)D in Serum* <*30 ng/mL (*<*75 nmol/L)*

If the serum 25(OH)D concentration is suboptimal, the previous treatment regimen, intake, dosage, and compliance should be verified. If compliance is adequate, adjusting the dose of cholecalciferol or switching to calcifediol when necessary is recommended.

*Concentrations of 25(OH)D in Serum 30–60 ng/mL (75–150 nmol/L):*

If serum 25(OH)D concentrations are stable at optimal sufficiency values, the prescribed regimen should be continued after verifying that compliance is adequate.

*Concentrations of 25(OH)D in Serum 60 to 100 ng/mL (150 to 250 nmol/L)*

Verify if the previous treatment regimen was appropriate and correct accordingly (dosage, compliance, type of preparation). Adjust the dose to achieve serum 25(OH)D concentrations between 30 and 60 ng/mL (75–150 nmol/L).

*Concentration of 25(OH)D in serum* > *100 ng/mL (*> *250 nmol/L):*

Levels above 100 ng/mL (> 250 nmol/L) of 25(OH)D may be associated with a higher risk of toxicity, so it is recommended to adjust intake, dosage, compliance, and/or the type of preparation used [30].

In case of suspected vitamin D intoxication, therapy should be discontinued immediately, and serum calcium, urinary calcium, and serum 25(OH)D concentration should be evaluated [41].

Vitamin D intoxication is defined as serum 25(OH)D concentrations > 100 ng/mL (> 250 nmol/L), accompanied by hypercalcemia, hypophosphatemia, hypercalciuria, and suppression of PTH.

After achieving normocalcemia, normocalciuria, and target 25(OH)D concentrations $\le$60 ng/mL ($\le$150 nmol/L), and excluding vitamin D hypersensitivity, prophylactic intake or therapeutic intervention can be resumed.

After six weeks of therapy discontinuation, serum 25(OH)D concentration should be reevaluated.

## Discussion

This recommendation aligns closely with global standards, as anticipated, given that it is based on current scientific evidence. However, two key factors distinguish this joint position and make it particularly impactful for improving healthcare practices in Mexico. Firstly, its emphasis on feasibility ensures that the recommendations can be applied nationwide, regardless of varying healthcare settings and resource availability. Secondly, the collaborative consensus-building process involving leading medical societies lends considerable strength to the position. A unified voice from medical leaders holds significant sway in shaping health policy decisions related to vitamin D supplementation.

An essential aspect of these recommendations is prioritizing feasibility to ensure the primary goal is attainable. For example, when determining 25(OH)D levels in a patient, the emphasis is placed on achieving the result rather than specifying the method used. Instead of mandating a particular technology known for its accuracy, the focus is on allowing patients to be evaluated using the available technology in their clinical environment. This approach acknowledges the significant heterogeneity of resources within the nation.

By prioritizing feasibility, these recommendations aim to ensure that all patients, regardless of their healthcare setting or available resources, have access to essential diagnostic evaluations. This approach promotes equity in healthcare and increases the likelihood of widespread implementation and adherence to guidelines. Furthermore, by adopting a flexible approach to diagnostic methods, healthcare providers can adapt to varying clinical contexts and resource constraints, ultimately improving patient care outcomes. This pragmatic approach aligns with the overarching goal of delivering high-quality, evidence-based care while recognizing and addressing the real-world challenges faced in healthcare delivery.

Through a systematic and evidence-based approach, we have endeavored to standardize guidelines, enhance healthcare delivery, and ultimately improve patient outcomes. Our journey began with a thorough assessment of the heterogeneity in vitamin D supplementation practices, analyzing the complexities and challenges inherent in clinical decision-making. Drawing upon the expertise of diverse stakeholders and leveraging robust methodologies such as systematic literature reviews and consensus-building techniques, we meticulously crafted a joint position that reflects the latest scientific evidence, clinical best practices, and the collective wisdom of the medical community.

Central to our approach was transparency, ensuring clarity regarding available evidence, limitations, and areas necessitating further research. By acknowledging the provisional nature of our recommendations and fostering an environment of continuous evaluation and refinement, we remain committed to advancing the science of vitamin D supplementation while adapting to emerging knowledge and clinical insights.

Moreover, our joint position underscores the importance of inclusivity, recognizing the diverse perspectives and experiences that shape clinical decision-making. By engaging with healthcare professionals, stakeholders, and the public, we sought to ensure that our recommendations are pragmatic, feasible, and reflective of the varied contexts in which they will be implemented. Implementing our joint position promises to enhance vitamin D supplementation practices’ consistency, quality, and effectiveness across Mexico. By providing clear guidance for healthcare providers, promoting evidence-based decision-making, and fostering a culture of continuous improvement, we aim to elevate the standard of care and promote population health.

This collective advocacy influences supplementation protocols and informs public health initiatives and funding allocation strategies. By leveraging their expertise and influence, medical societies can effectively endorse policies promoting optimal vitamin D intake and advancing population health outcomes.

This consensus stands out for several strengths contributing to its robustness and applicability in the medical field. A diverse panel was formed with representatives from various clinical and surgical specialties, composed of highly academic professionals. This diversity enriches the perspective on the indications and contraindications of vitamin D, providing a comprehensive and applicable view in different medical areas. The participation of various national medical organizations reinforces penetration into patient care sectors.

The methodology used in developing this document minimizes the risk of bias, ensuring the quality and objectivity of the recommendations. It is important to note that this document represents the first consensus on vitamin D in Mexico and is a joint position statement. The generated recommendations follow international standards and apply to both healthy adults and those facing various pathologies. Furthermore, the importance of making therapeutic decisions with patients and caregivers and respecting their preferences is acknowledged.

However, this consensus also presents some limitations. Evidence based on randomized clinical trials in the Mexican population is scarce, which may influence the robustness of some recommendations. Although upper limits of 25(OH)D up to 100 ng/mL are established, current evidence suggests the possibility of lower limits, although there is inconsistency in the data. Regarding dosing, the lack of compelling evidence on the best option in various clinical scenarios led to adaptation to the available formulations in the country. Although pragmatic, this approach may not address possible future options that could emerge in the market.

### Conclusion

In conclusion, the development of Mexico’s national joint position on vitamin D supplementation is a significant landmark in our ongoing efforts to address unwarranted variations in clinical practice. Through the collaboration of diverse stakeholders and robust methodologies, we have developed recommendations grounded in scientific evidence, clinical best practices, and the collaborative understanding of the medical community. We aim to ensure these recommendations are effectively implemented across healthcare settings, improving healthcare delivery and advancing public health outcomes. As we move forward into the implementation and evaluation phase, we remain committed to scientific rigor, collaboration, and patient-centered care.

By acknowledging our consensus’s strengths and limitations, we position ourselves to continuously refine and adapt our recommendations, better aligning them with the evolving needs of patients and healthcare providers. Through ongoing evaluation and sustained collaboration, we endeavor to realize the full potential of our collaborative efforts in fortifying healthcare delivery and fostering the population’s well-being.

## Data Availability

This position paper does not report original data. No datasets were generated or analyzed during the current study.

## Declarations

### Disclaimer

Although most clinical recommendations are intended to be applied nationwide, social disparities may prevent full implementation in certain regions. However, these recommendations can serve as a valuable reference for public health agencies and policymakers, guiding the improved allocation of health resources based on a solid national medical position. Furthermore, feedback from clinicians and healthcare administrators may lead to future revisions and updates as necessary.

### Endorsements

The following societies endorse this consensus report: College of Internal Medicine of Mexico (CMIM), Mexican Academy of Geriatrics (AMG), Mexican Association for the Study of Climacteric (AMEC), Mexican Association of Bone and Mineral Metabolism (AMMOM), Mexican College of Orthopedics and Traumatology (CMO), Mexican College of Rheumatology (CMR), National College of Geriatric Medicine (CONAMEGER), Mexican Federation of Obstetrics and Gynecology Colleges (FEMECOG), Mexican Federation of Orthopedics and Traumatology Colleges (FEMECOT).
